# Surface-guided breathing signal integration in breathing-adapted intelligent 4D computed tomography: prototype implementation and comparison with an infrared marker-based system

**DOI:** 10.1016/j.phro.2025.100848

**Published:** 2025-10-10

**Authors:** Niklas A. Lackner, Torsten Moser, Julian Young, Jannis Dickmann, Volker That, Christian Hofmann, Andre Karius, Mushawar Ahmad, Oliver J. Ott, Florian Putz, Rainer Fietkau, Christoph Bert, Juliane Szkitsak

**Affiliations:** a Department of Radiation Oncology, Universitätsklinikum Erlangen, Friedrich-Alexander-Universität Erlangen-Nürnberg, Universitätsstraße 27, 91054 Erlangen, Germany; b Comprehensive Cancer Center Erlangen-EMN (CCC ER-EMN), Erlangen, Germany; c Comprehensive Cancer Center Alliance WERA (CCC WERA), Erlangen, Germany; d Bavarian Cancer Research Center (BZKF), Erlangen, Germany; e Vision RT LTD, London, United Kingdom; f Siemens Healthineers AG, Forchheim, Germany

**Keywords:** 4DCT, Experimental prototype study, Breathing motion, Surface-guidance

## Abstract

**Background and purpose:**

Breathing-adapted intelligent four-dimensional computed tomography (i4DCT) reduces motion artifacts during irregular breathing using real-time surrogate signal analysis to control X-ray timing. Current implementations rely on infrared (IR) markers or pressure belts. Surface-guided radiation therapy (SGRT), a widely used markerless technique in radiotherapy, has not been evaluated for direct i4DCT integration. This study tested SGRT for i4DCT acquisition in a phantom setup and benchmarked it against an infrared marker-based system.

**Materials and methods:**

Phantom measurements with clinically relevant regular and irregular breathing signals were performed on a prototype CT scanner with direct SGRT control. To improve SGRT-based surrogate accuracy, a table motion correction profile was empirically modeled, and a vendor-specific prediction algorithm was implemented. Surrogate signal accuracy, latency, and motion reconstruction accuracy were compared between SGRT and an established IR system using amplitude- and phase-based reconstructions.

**Results:**

SGRT exhibited an absolute latency of ∼ 63  ms, compared to ∼ 23  ms for the IR system. Across regular and irregular breathing signals, SGRT breathing signals showed Root Mean Square Error (RMSE) values up to ∼ 1.5  mm, while correlation with the ideal signal remained high (r = 1). Tumor center-of-mass deviations in amplitude-based reconstructions reached 1.5  mm without prediction at 20 breaths-per-minute and 15  mm amplitude, but reduced to < 0.5  mm with 50  ms prediction. Both amplitude- and phase-based reconstructions showed improved agreement with ideal motion when prediction was applied, with phase-based reconstructions yielding better accuracy.

**Conclusions:**

These findings support SGRT as a clinically viable non-contact alternative to IR tracking in i4DCT, especially when combined with motion correction and predictive modeling.

## Highlights

• Surface guided signal used for the first time to control intelligent 4D computed tomography.
• Motion correction and prediction algorithm improved signal accuracy.
• Breathing curves from surface tracking closely matched ideal motion pattern.
• Tumor motion error reduced from 1.5 mm to below 0.5 mm with prediction.
• Contactless tracking preserved image quality even under irregular breathing.

## Graphical abstract

**Figure:**
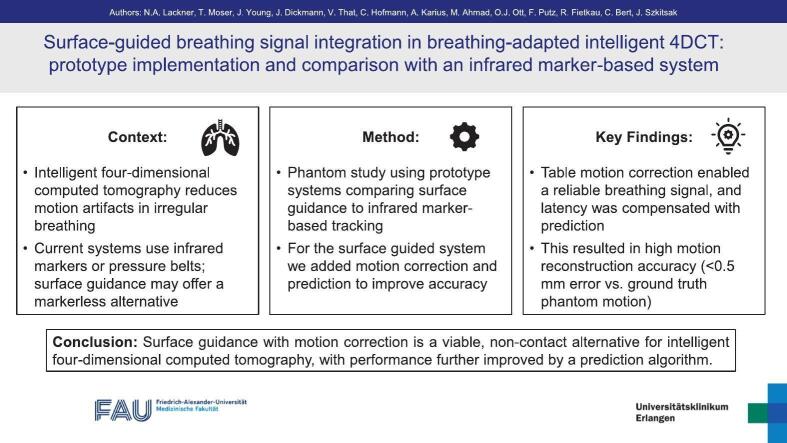

## 1 Introduction

Breathing motion during radiotherapy challenges imaging accuracy and treatment delivery, particularly for thoracoabdominal tumors [1,2]. Four-dimensional computed tomography (4DCT) characterizes motion by acquiring images at different breathing phases [[3], [4], [5], [6]]. However, conventional 4DCT relies on retrospective binning of projection data [[7], [8], [9]], which can cause motion artifacts in the presence of irregular breathing. These issues impact tumor delineation and may necessitate larger treatment margins or repeating imaging [[10], [11], [12], [13]].

In response, intelligent 4DCT (i4DCT) was developed to adapt 4DCT scans prospectively to the patient’s breathing in real-time [[14], [15], [16]], similar to earlier prospective approaches [[17], [18], [19]]. By analysing the breathing signal in real time during a sequential data acquisition, i4DCT triggers X-ray-on/-off acquisition only when certain conditions for breathing state and data completeness are met, enabling complete data capture even for irregular breathing [[20], [21], [22], [23]]. This breathing-synchronized approach improves 4D imaging quality over conventional spiral 4DCT, reducing data insufficiency and motion artifacts without a relevant increase in dose [24,25]. However, i4DCT imposes strict requirements on real-time surrogate signal integration.

Current implementations of the i4DCT algorithm on commercial CT scanners support only infrared (IR) reflective marker-based and pressure sensor belt systems, both of which have inherent limitations. IR marker-based systems require a dedicated marker block that must be precisely positioned for reliable detection, while pressure belts involve cumbersome setup and rely on patient compliance. Surface-guided radiation therapy (SGRT) offers a markerless alternative [[26], [27], [28], [29]], tracking the patient’s skin surface to derive a breathing signal [[30], [31], [32]]. While SGRT is widely established for patient positioning, intrafractional motion monitoring during treatment delivery, and increasingly used during CT imaging in radiation oncology [31], its practical feasibility for integration into i4DCT acquisition workflows remains unproven. Prior feasibility studies identified SGRT as a potential surrogate for i4DCT, but were limited to simulations and experiments without direct scanner control. One study additionally showed that uncorrected table motion and a 45  ms signal delay can impair i4DCT accuracy when using SGRT, highlighting the need for table motion modeling and breathing signal prediction [33,34].

To bridge this gap, we evaluated the experimental integration of SGRT into i4DCT using a prototype CT scanner that allowed real-time control by an SGRT system via a breathing gating interface. Phantom measurements compared SGRT-based acquisitions with an established IR marker system. To improve SGRT surrogate accuracy, we implemented table motion correction and a vendor-specific prediction algorithm. The study quantified SGRT signal accuracy, assessed latency effects on motion reconstruction accuracy, and evaluated whether markerless surface guidance fulfilled i4DCT requirements in a controlled experimental setting.

## 2 Materials and methods

### 2.1 Research implementation of intelligent 4DCT scanning

A prototype multi-slice CT scanner (SOMATOM go.Open Pro, pre-release syngo.CT VB20), the Direct i4D acquisition algorithm [[13], [14], [15]], and the non-clinical reconstruction software ReconCT (v18.0_prerelease), all developed by Siemens Healthineers AG, Germany, were used in this study. The scanner featured a respiratory gating interface enabling real-time data exchange with both surface-guided and marker-based surrogate systems. 4DCT scans were acquired in sequential scanning mode. The CT scanner was equipped with 64 detector rows at 0.6 mm width, defining the total collimation. The 4DCT scans were acquired in sequential scanning with a fixed table increment of 0.9 × (64 × 0.6 mm). Amplitude- and phase-based 4DCT reconstructions were generated, each yielding 20 3D images. Images were reconstructed using a 768 × 768 matrix, 1  mm slice thickness, 500  mm field-of-view, and kernel OR40f (medium-smooth, fine‑texture).

### 2.2 Surrogate systems

Three optical surrogate systems were used in this study: the Respiratory Gating for Scanners (RGSC) system (v1.1.25.0, Varian Medical Systems, USA), a research version of SimRT (based on v7.2, Vision RT, UK), and Polaris Spectra (vIL-1070101 R5, Northern Digital Inc., Canada).

RGSC employs a table-mounted IR camera to track a passive marker block, deriving the breathing signal from the vertical displacement of the block.

SimRT, a ceiling-mounted surface-guided system, uses stereo photogrammetry to track a 5 × 5 cm^2^ region of interest (“patch”). To account for table motion, SimRT applies a model based on the measured table travel profile, using a fixed 34.5 mm increment for patch propagation. To mitigate system latency, SimRT employs an experimental real-time prediction algorithm, currently developed for non-clinical use, which uses a proprietary model based on polynomial extrapolation of recent breathing amplitudes to predict future signal values by a defined temporal offset. A previous study [33] reported a relative latency of ∼ 45  ms between the IR marker system and the surface-guided setup, but absolute end-to-end latencies of the surface-guided system, specific to our experimental configuration, were unknown at the time of the measurements. To address this, fixed prediction offsets of 50 ms and 100 ms were applied: the former based on the previously observed latency, and the latter to test the effect of a stronger predictive correction beyond the reported latency. SimRT was operated in a research configuration that enabled real-time streaming to the CT scanner’s gating interface.

Polaris Spectra, mounted on a tripod beside the CT table, served as a reference system for latency measurements and to acquire the table motion correction profile. This optical tracking system has a known baseline latency of 16.6 ± 1 ms [35], which was accounted for in all latency analyses. Like RGSC, Polaris tracks IR markers using a positional sensor. Using NDI’s 6D Architect software (v3.02.04), both the RGSC marker block—used to track the breathing signal—and a custom 3D-printed reference object affixed to the CT table—used to capture the table motion profile—were tracked simultaneously. The acquired table motion profile was used to correct SimRT-derived signals (see Supplementary Material, Fig. S1). Polaris was not included in system performance benchmarking.

### 2.3 Phantom measurements in a prototype setup

For all measurements, we used a spherical tumor insert (Ø 2  cm) mounted within a dynamic thorax phantom (CIRS model 008A, Sun Nuclear, USA, Supplementary Material Fig. S2). The insert and surrogate platform were moved according to a predefined breathing waveform, with a motion accuracy of ± 0.1 mm [36]. To enable detection by the surface tracking system, the RGSC marker block was covered in white tape. The marker block was positioned atop the moving surrogate platform of the dynamic thorax phantom. The SimRT breathing patch was placed centrally on the RGSC marker block. All components, including the chest plate and tumor insert, were mechanically coupled and moved synchronously. Additionally, the 3D-printed reference object was positioned on the table at the same longitudinal position as the RGSC marker block for tracking using the Polaris.

Typical breathing and tumor motion amplitudes in the anterior-posterior (A–P), superior-inferior (S–I), and lateral-medial (L–M) directions were estimated from a retrospective cohort of 370 upper-abdominal and thoracic patients treated at our institution. To capture the range of breathing irregularities encountered in 4DCT imaging, we programmed the motion phantom with various synthetic cos⁶-based breathing signals

(1) $$\zeta \left(t\right)=\;A_{p2p}*\cos^{6}\left(\frac{2\pi t}{T_{cycle}}\right)\;$$

where $A_{p2p}$ denotes the peak-to-peak amplitude and $T_{\mathrm{cycle}}$ the breathing cycle duration. In addition to variations of cos⁶-based reference waveforms as defined in Eq. (1), an irregular waveform and patient-derived breathing traces recorded with the RGSC system during routine clinical procedures were employed to replicate clinically observed breathing signals. A set of five representative breathing traces was selected to reflect clinically relevant variability in breathing signals, covering breathing rates (BRs) between 6 and 20 breaths per minute (BPM) and peak-to-peak amplitudes between 5 and 25  mm. These amplitudes correspond to the vertical motion of the external marker block mounted on the phantom surface. Each breathing trace was combined with one of three independently defined internal tumor motion scenarios, set to 5/1/1 mm, 15/2/2 mm, and 25/5/5 mm in the S–I, A–P, and L–M directions, respectively. All measurements were repeated three times per configuration. The complete measurement matrix is provided in the Supplementary Material Table S1, and a photograph of the experimental setup is shown in the Supplementary Material Fig. S3.

### 2.4 Data analysis

Breathing signals were exported from RGSC and SimRT containing acquisition time, amplitude values, and X-ray-on/-off status. Polaris data acquisition was performed using Python 3.7.14 and the open-source toolkit *SciKit-Surgery* [37]. Data analysis was conducted in MATLAB (R2019b, The MathWorks Inc., Massachusetts, USA). All signals were resampled to the same time grid resolution (20 ms) and truncated to a common time window, spanning from 20 s before the first X-ray-on to 10 s after the last X-ray-on. Amplitude values were converted to millimeters based on each system’s scaling. All breathing signals were smoothed using a moving average filter with a 100  ms window (5 samples at 20  ms sampling interval), and baseline-normalized by subtracting the minimum value between the first two local maxima. The analysis was structured into the following key analyses:

**System Latency Measurement:** To assess system latency, we applied regular sinusoidal motion

(2) $$\zeta \left(t\right)=A_{p2p}*\sin\left(\frac{2\pi t}{T_{cycle}}\right)$$

in ten measurement runs. Each run included brief manual motion interruptions—implemented as sudden stops followed shortly by restarts—to create abrupt transitions that served as reference points for clearly identifying the subsequent motion maxima (Supplementary Material Fig. S4). Simultaneous signal acquisition was performed for SimRT, RGSC, and Polaris (used as the reference). All systems transmitted real-time data to a single workstation via RS-485 to USB (SimRT and RGSC) or USB (Polaris), using the same RS-485 protocol intended for scanner communication. Recorded signals were smoothed as described above and fitted to ideal sinusoids, and latency was quantified by comparing the timing of corresponding local maxima immediately following the abrupt transitions.

**Breathing Signal Evaluation in Phantom Measurements:** Fixed prediction offsets of 50  ms and 100  ms, applied as described in Section 2.2 were tested for regular breathing signals of 12 BPM/15 mm and 20 BPM/15 mm. All signals were temporally aligned and truncated to a common window (20  s before the first and 10  s after the last X-ray-on). The breathing signals were compared against reference signals, typically based on idealized cos⁶ functions (see Supplementary Material Table S1), except for irregular signals where SimRT and RGSC were compared directly, using the BR in BPM, the root mean square error (RMSE), mean absolute error (MAE), and Pearson correlation coefficient (r). Let $x_{i}$ and $\;y_{i}$ denote the amplitude values of the measured and reference signals, respectively, at time point i and let *N* be the number of samples. The metrics were calculated as

(3) $$BR=\;\frac{60}{T_{\mathrm{cycle}}}\;$$

(4) $$RMSE\;=\;\sqrt{\left(\begin{array}{c} \frac{1}{N} \end{array}\right)\sum_{i=1}^{N}{\left(x_{i}-\;y_{i}\right)}^{2\;}\;}$$

(5) $$MAE\;=\;\left(\begin{array}{c} \frac{1}{N} \end{array}\right)\sum_{i=1}^{N}\left|x_{i}-\;y_{i}\right|\;and$$

(6) $$r\;=\frac{\sum_{i=1}^{N}\left(x_{i}-\;\overset{¯}{x}\right)\left(y_{i}-\;\overset{¯}{y}\right)}{\sqrt{\sum_{i=1}^{N}{\left(x_{i}-\;\overset{¯}{x}\right)}^{2}}\sqrt{\sum_{i=1}^{N}{\left(y_{i}-\;\overset{¯}{y}\right)}^{2}}}$$

here, $\overset{¯}{x}$ and $\overset{¯}{y}$ denote the mean values of x and y, respectively. To assess breathing motion quality during distinct phases (inhalation (Inh) and exhalation (Exh)) of the breathing cycle, phase-specific RMSE and r values were computed for each cycle and averaged across all complete breathing cycles within a session. Inhalation was defined as the interval between a local minimum and the subsequent maximum, and exhalation as the interval from that maximum to the next minimum, as detected on the reference signal.

**Motion Reconstruction Accuracy in Phantom Measurements:** Motion reconstruction accuracy was evaluated using amplitude- (AB) and phase-based (PB) reconstructions. Center-of-mass (COM) positions of the spherical tumor insert were extracted from each of the twenty reconstructed breathing phases and analysed along the L-M, A-P, and S-I axes. For AB reconstructions, the reference motion trajectory was defined as a piecewise linear path from Exh100 to Inh0 and then to Inh100. For PB reconstructions of regular breathing signals, the corresponding reference curves were based on idealized cos⁶ functions. For PB reconstructions of irregular breathing signals, SimRT and RGSC were compared directly. For each phase bin, COM values from the repeated scans within a measurement set were averaged, and the corresponding standard deviations (SDs) were calculated to assess inter-scan variability.

## 3 Results

### 3.1 System latency measurement

The timing of local maxima following each sudden stop was used for latency estimation. Across ten repeated motion cycles, system latencies relative to the Polaris reference were (45.9 ± 7.8  ms) for SimRT and (6.4 ± 3.0) ms for RGSC (mean ± SD). Taking into account the Polaris baseline delay of 16.6 ± 1 ms, this corresponds to absolute latencies of approximately 63  ms for SimRT and 23  ms for RGSC.

### 3.2 Breathing signal evaluation in phantom measurements

**Fig. 1:**
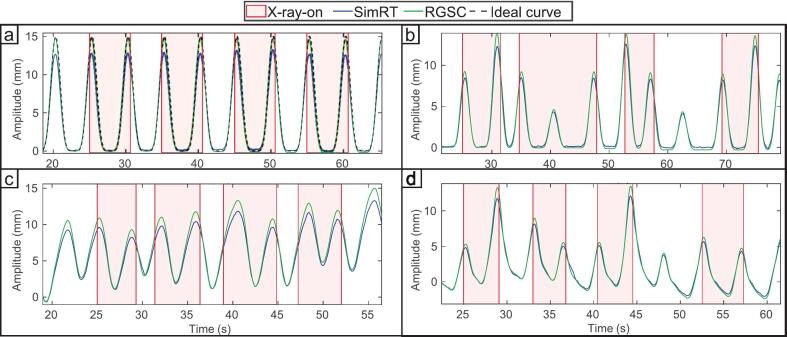
Representative breathing signals acquired with the SimRT and RGSC systems (see Supplementary Material Table S1 for parameter definitions). (a) Regular breathing signal recorded in phantom measurements; a dashed ideal reference signal is also shown. (b) Irregular breathing signal in phantom measurements. (c) Regular breathing signal from a patient measurement. (d) Irregular patient breathing signal. Red shaded regions indicate periods of X-ray activation. All breathing signals were baseline-normalized to the first local minimum. The x-axis (time) is shared across all subplots. Note: The ideal reference signal is shown only in (a); irregular and patient signals were compared directly without a reference. (For interpretation of the references to colour in this figure legend, the reader is referred to the web version of this article.)

Across all regular breathing signals, RGSC consistently showed minimal deviation from the reference signals, with RMSE and MAE values typically below 0.2  mm and r = 1.0, indicating excellent agreement. In contrast, SimRT exhibited higher RMSE values (up to 1.5  mm), particularly at larger amplitudes (25 mm) or during irregular breathing, although the correlation remained high with r = 1. The application of the SimRT prediction algorithm had a negligible effect on breathing signal quality. RMSE and MAE values remained nearly unchanged when predicting 50  ms and 100  ms ahead. Phase-specific metrics, such as r for inhale and exhale, consistently remained at r = 1.0. This indicates that the prediction algorithm, while compensating for system latency, preserved the accuracy of the breathing signal during both regular and irregular breathing signals. Fig. 1 illustrates representative breathing signals for different scenarios, highlighting both temporal alignment and amplitude accuracy across methods. For detailed metrics across all tested breathing rates, amplitudes, and irregular breathing signals, refer to Table 1.

**Table 1:** Aggregated breathing signal metrics (mean ± standard deviation) comparing RGSC and SimRT systems across all scan sessions. Breathing signals were acquired with varying breathing rates (BR, in bpm) and amplitudes (AMP, in mm). Metrics include the root mean square error (RMSE), mean absolute error (MAE), Pearson correlation coefficient (r), as well as phase-specific RMSE and r for inhalation and exhalation. Values represent agreement between the respective breathing signals and ideal reference signals during intelligent 4DCT acquisitions. Grey-highlighted cells indicate absolute differences between RGSC and SimRT.

Abbreviations: Exh = exhalation; Inh = inhalation; pred50 = prediction algorithm predicting 50  ms ahead; pred100 = prediction algorithm predicting 100  ms ahead; irregPha = irregular phantom signal; regPat = regular patient signal; irregPat = irregular patient signal.

### 3.3 Motion reconstruction accuracy evaluation in phantom measurements

To assess motion reconstruction accuracy, both PB and AB reconstructions were applied across all phantom scan settings. COM trajectories of the tumor insert were extracted along the S-I, A-P, and L-R directions. Deviations in the A-P and L-R directions were smaller than those in the S-I direction, which exhibited the largest motion amplitudes. However, when normalized to the peak-to-peak amplitude of each direction, relative deviations in A-P and L-R were similar to those in S-I and typically remained below 10 % compared to reference values during high-motion phases. As the S-I direction was the dominant motion axis in this study, it provided the highest sensitivity for evaluating motion accuracy. Therefore, the results in this section primarily focus on the S-I direction.

Phase-based reconstructions generally outperformed amplitude-based reconstructions in both the RGSC and SimRT systems. For example, at 12 BPM/15  mm, RGSC achieved − 0.1 ± 0.1  mm (PB-Exh) and 0.5 ± 0.2  mm (AB-Exh) COM deviation, while SimRT showed 0.1 ± 0.1  mm (PB-Exh) and 0.9 ± 0.3  mm (AB-Exh) COM deviation. Similar behaviors were observed across other acquisition settings, with AB consistently showing larger deviations due to its increased sensitivity to irregular or shifting breathing behaviour. A comprehensive summary of all the results with the varying breathing rate and amplitude combinations is provided in Table 2.

**Table 2:** Overview of center of mass (COM) differences in the superior-inferior (S-I) direction (in mm, shown as mean ± standard deviation) for different breathing motion settings, comparing measurements obtained with RGSC and SimRT. Results of the different systems are compared against ideal signals: for amplitude-based reconstructions, a linear reference from Exh100 to Inh0 and from Inh0 to Inh100 was used; for phase-based reconstructions, ideal cos⁶ breathing signals were used. Breathing signals were acquired with varying breathing rates (BR, in bpm) and amplitudes (AMP, in mm). Exhalation (Exh) values were inverted ( *-1) so that positive values indicate a lag in inferior-superior COM position relative to the ideal reference signal. Grey-highlighted cells indicate absolute differences (mean ± standard deviation) between the RGSC system and SimRT.

Abbreviations: AB = amplitude-based reconstruction; PB = phase-based reconstruction; Exh = exhalation; Inh = inhalation; pred50 = prediction algorithm predicting 50  ms ahead; pred100 = prediction algorithm predicting 100  ms; ahead; irregPha = irregular phantom; regPat = regular patient signal; irregPat = irregular patient signal. “not const.” non-constant breathing signals.

Across all conditions, RGSC consistently yielded the lowest deviations from the ideal motion trajectory. SimRT without prediction showed larger offsets, particularly during exhalation, with deviations reaching up to 1.5 ± 0.5  mm (AB-Exh) at 20 BPM/15  mm. The application of the prediction algorithm substantially improved COM accuracy. At 12 BPM/15  mm, SimRT prediction reduced AB-Exh deviations from 0.9 ± 0.3  mm to 0.3 ± 0.1  mm (50  ms) and − 0.3 ± 0.2  mm (100  ms). At 20 BPM/15  mm, AB-Exh deviations dropped from 1.5 ± 0.5  mm to 0.4 ± 0.2  mm (50  ms) and − 0.3 ± 0.3  mm (100  ms). Notably, AB deviations for SimRT with 50  ms prediction were lower than for RGSC in both exhalation and inhalation phases at 20 BPM/15 mm (AB-Exh: 0.4 ± 0.2  mm vs. 0.9 ± 0.3  mm; AB-Inh: 0.3 ± 0.1  mm vs. 0.4 ± 0.2  mm), confirming effective compensation of latency-induced tracking offsets.

**Fig. 2:**
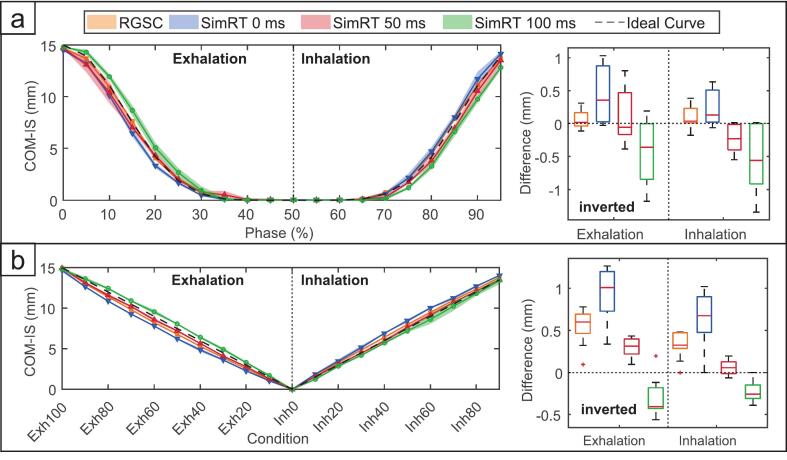
The center-of-mass position along inferior-superior (COM-IS) axis is plotted against reconstructed breathing phases (left) and corresponding differences to the ideal motion profile (12 BPM, 15 mm) are shown as boxplots (right). Exhalation values in the difference plots were inverted so that positive differences indicate a lag relative to the ideal signal. (a) Phase-based reconstruction: COM-IS trajectories across all systems closely follow the ideal motion. Boxplots on the right show that SimRT without prediction lags behind the reference during exhalation. A 50  ms prediction effectively reduces this lag and improves motion reconstruction accuracy, while a 100  ms prediction results in overcompensation. (b) Amplitude-based reconstruction: A similar behavior is observed, with the 50  ms prediction algorithm effectively minimizing deviations, particularly during exhalation. In contrast, the 100  ms prediction overcorrects, suggesting that prediction closer to the system’s actual latency offers the most effective compensation for motion tracking delays.

Fig. 2 further illustrates these findings for the 12 BPM/15  mm condition, showing COM-IS positions across breathing phases and corresponding deviations from the ideal signal. The boxplots demonstrate a clear trend: SimRT without prediction exhibits a systematic lag, particularly during exhalation. While both 50  ms and 100  ms prediction settings reduce this latency, the 50  ms setting achieves closer alignment with the ideal motion trajectory, whereas 100  ms prediction shows a tendency to overcompensation.

## 4 Discussion

This prototype study evaluated the integration of surface guidance into i4DCT with real-time surrogate-based acquisition control. Latency, breathing accuracy, and motion reconstruction were assessed and compared against a clinical infrared system and phantom ground truth. Key findings include measurable latency differences, consistent breathing signal quality, and comparable motion reconstruction performance between systems under controlled conditions.

In a previous simulation study using experimental clinical data, we showed that i4DCT with surface guidance could match marker-based infrared surrogates if table motion correction and latency were addressed [33]. SimRT showed higher latency than RGSC after accounting for Polaris baseline delay [35]. Even moderate delays can impair temporal accuracy during rapid breathing, affecting phase assignment and motion binning. While no formal threshold exists, prior work emphasizes minimizing delay to preserve motion fidelity [1,33].

SimRT and RGSC signals showed strong agreement under all conditions. Despite amplitude differences, both responded linearly with excellent correlation. As i4DCT binning relies on relative, not absolute amplitude, correlation is more clinically relevant than RMSE, supporting SGRT use even with mismatches. Table motion correction was reliable, and deviations are unlikely to compromise reconstruction accuracy or internal target volume (ITV) generation [38,39]. The fixed table feed correction of 34.5  mm differed only minimally from the nominal 34.56  mm, which was negligible for the short scan lengths investigated; however, longer acquisitions may amplify this effect, and future corrections should employ the exact table profile.

PB binning yielded more accurate motion reconstructions than AB binning, particularly under regular breathing. Though AB binning is often preferred clinically for irregular breathing patterns [22,25,40], PB was more stable under idealized conditions. AB was more sensitive to latency-induced shifts during rapid transitions. A 50  ms prediction offset matched SimRT’s delay and improved accuracy, occasionally outperforming RGSC. While submillimetric deviations may seem minor, they become relevant in high-precision treatments like stereotactic body radiation therapy (SBRT), where small misalignments risk underdosage or dose shifts [24,41].

Respiratory irregularities in BR and amplitude challenge conventional 4DCT, which assumes regular breathing. Prior studies showed such irregularities cause artifacts and inaccurate reconstructions, better addressed by real-time strategies like i4DCT [24,25,42,43]. To ensure clinical relevance, phantom and patient signals here incorporated irregularities in both phase and amplitude. Literature and our clinical data show that external surrogate amplitudes typically range from 5 to 25 mm. Tumor motion can reach up to 25 mm in the S–I direction, substantially exceeding A–P and L–M components [28,44,45].

This study focused on moderate breathing amplitudes (5–25  mm), reflecting common clinical cases. Very low-amplitude signals (<5 mm), were not systematically assessed. Such signals are more noise-prone and can impair trigger accuracy [40], increasing the risk of surrogate failure. Rare cases of minimal external motion with substantial internal displacement have also been reported [46], underscoring the need to investigate internal–external correlation and robustness in low-amplitude scenarios.

This study was limited to a single CT scanner and a single SGRT system, which applied a vendor-specific prediction algorithm based on polynomial extrapolation. While suitable under regular breathing, such linear models often struggle with irregular breathing patterns [47]. Advanced methods like long short-term memory (LSTM) networks improve robustness, achieving RMSEs of ∼ 0.05 (normalized units) for 500  ms predictions and reducing errors by ∼ 20 % [48]. Still, deep learning may fail under abrupt shifts or erratic breathing [49]. Future implementations of SGRT-based i4DCT should explore adaptive or hybrid frameworks that combine machine-learning predictors, and patient coaching to improve latency compensation. Additionally, the applied table motion correction was system-specific and may require calibration for broader use. Although phantom experiments allowed precise technical evaluation, clinical validation is necessary to fully assess treatment accuracy.

This study demonstrates that SGRT is a robust, markerless surrogate for i4DCT when combined with table motion correction. Performance is further increased with latency compensation, supporting broader clinical adoption and enhancing flexibility in motion-guided acquisition. Future work should validate this approach clinically and explore integration with multi-vendor platforms to enable widespread implementation.

## 5 Declaration on Generative AI

Generative AI (OpenAI ChatGPT v-4o) was utilized for proofreading this manuscript. All AI-generated suggestions were carefully reviewed and edited by the authors to ensure accuracy and clarity. The authors take full responsibility for the final content.

## Declaration of competing interest

The authors declare the following financial interests/personal relationships which may be considered as potential competing interests: The Universitätsklinikum Erlangen and the Department of Radiation Oncology have institutional research grants with Siemens Healthineers AG and Vision RT Ltd, but not directly related to this project.
