# Supplementary material for: Surface-guided breathing signal integration in breathing-adapted intelligent 4D computed tomography: prototype implementation and comparison with an infrared marker-based system
Source: Phys Imaging Radiat Oncol. 2025 Oct 10;36:100848. doi: 10.1016/j.phro.2025.100848 (PMC12552975; doi:10.1016/j.phro.2025.100848)
Supplement: Supplementary Data 1 [file mmc1.docx]

# Supplementary Material


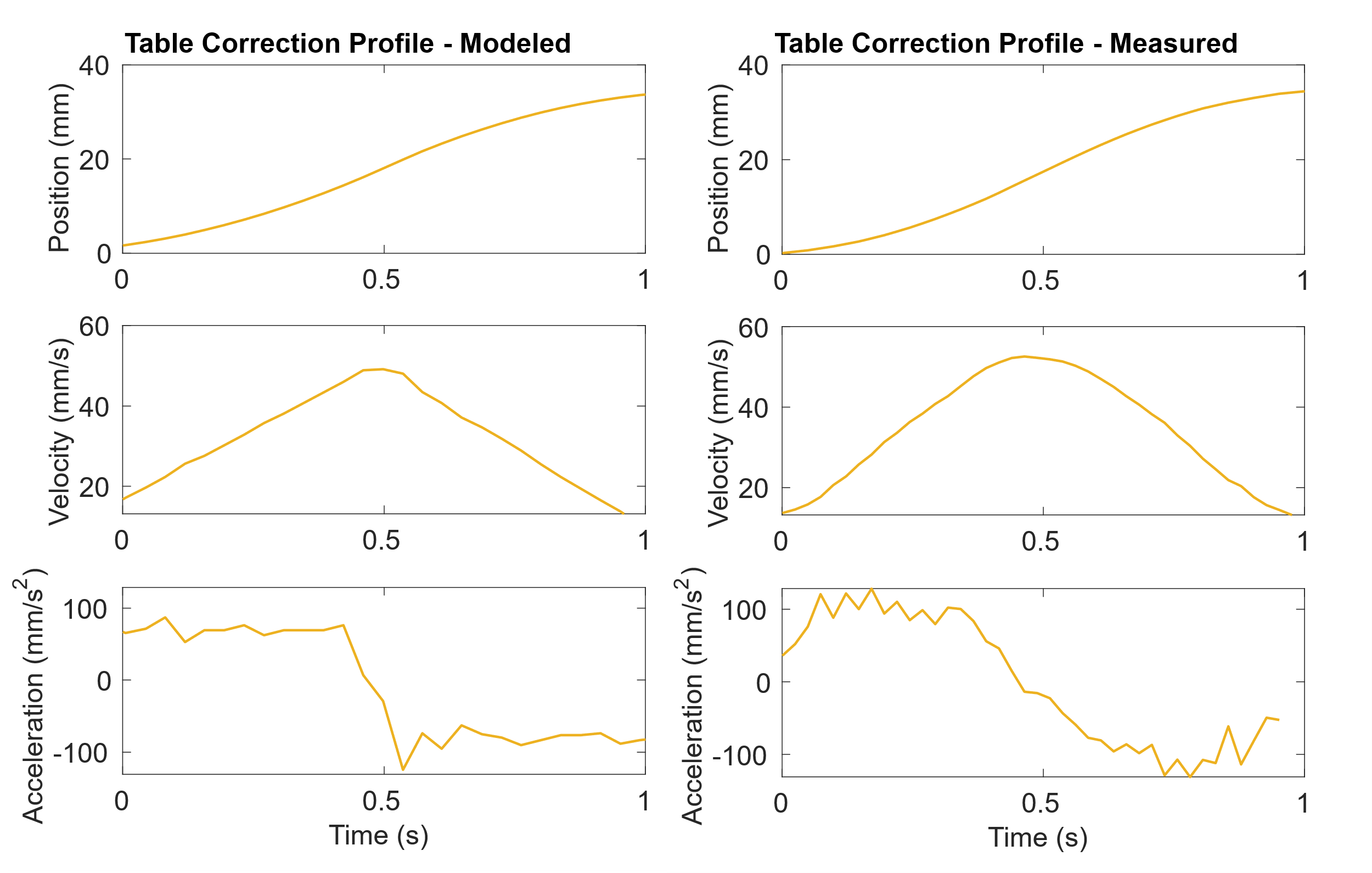


**Figure S1**. *Left: Axial table motion profile measured using the Polaris Spectra system during sequential scanning. The plots show the characteristic motion pattern of the table each time it advances to a new position, which remains consistent across all table movements during the i4DCT acquisition. Right: Empirically fine-tuned correction model implexmented in SimRT to match the measured motion profile. Empirically fine-tuned refers to the manual adjustment of motion characteristics (acceleration, deceleration, velocity, total table travel) to match the measured profile. The plots in each column show: (1) position over time, (2) velocity over time, and (3) acceleration over time, with velocity and acceleration derived from the position data.*


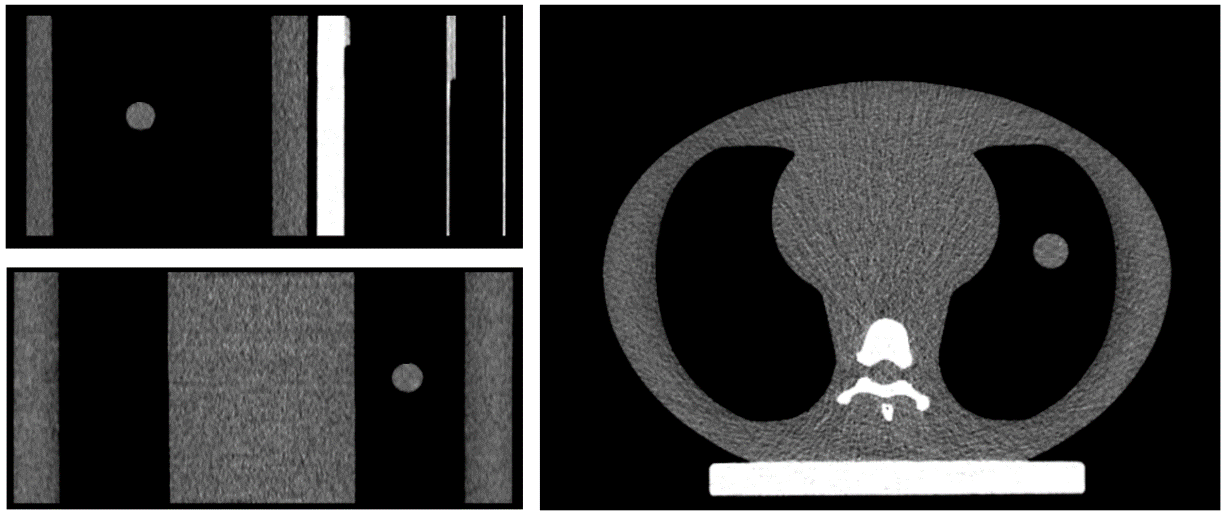


**Figure S2.** *Representative CT axial slice of a dynamic thorax phantom with lung compartments and a spherical tumor insert (visible in the right part of the image).*


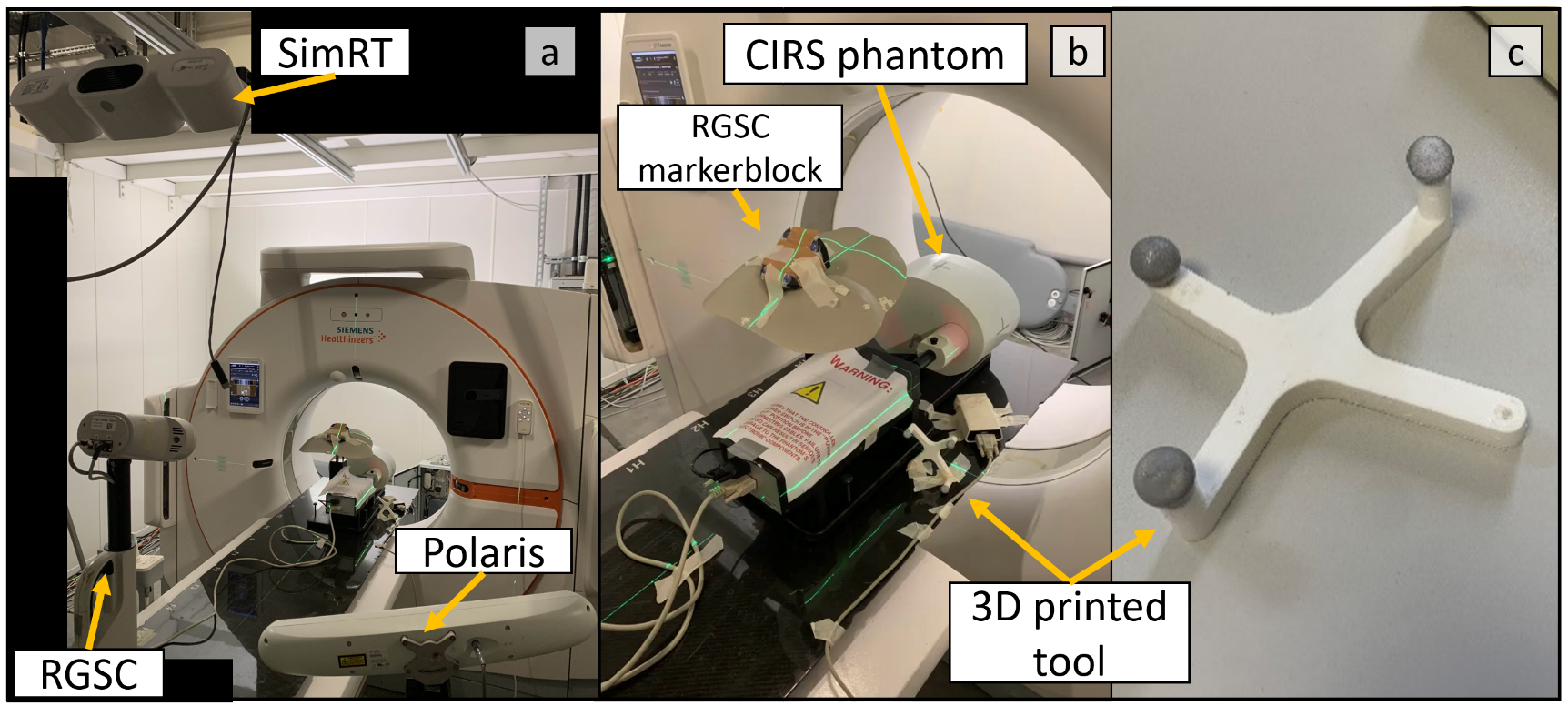


**Figure S3.** *Prototype measurement setup. a) Camera point of view showing the three surrogate systems: ceiling-mounted SimRT, table-mounted RGSC, and tripod-mounted Polaris system. b) Zoomed-in view of the phantom setup, including the CIRS dynamic thorax phantom, the RGSC marker block (partially covered with tape to enhance SGRT visibility), and the external surface phantom as well as the 3D printed tool.
c) Custom 3D printed tool attached directly to the CT table, designed to allow Polaris tracking of table motion independently.*


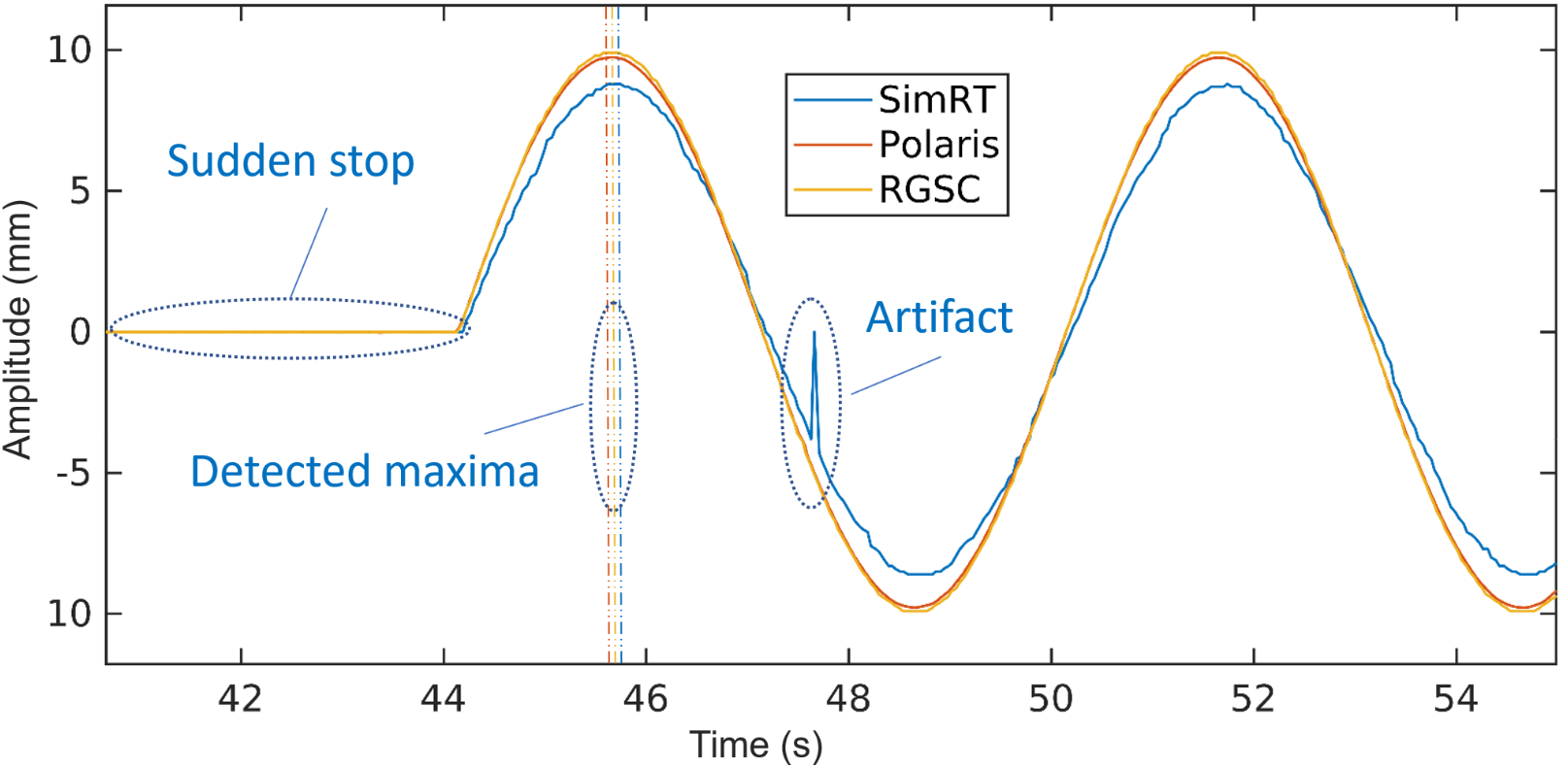


**Figure S4.** *Illustration of the method used for determining system latency based on abrupt motion interruptions (sudden stops) during controlled sinusoidal motion. Local maxima immediately following the stop are used as temporal landmarks for latency estimation across SimRT, Polaris (reference), and RGSC systems. Detected maxima were manually shifted in the plot to avoid overlap and improve clarity. Artifacts like those visible in the plot had no effect on latency determination and were not present in the RS-485 datastream used during CT experiments with the SGRT system.*

**Table S1*.*** *Overview of the parameters selected for the measurements with three-dimensional phantom movement. Each configuration was repeated three times with both RGSC and SimRT, except for predictive motion types, which were tested only with SimRT. Surrogate motion refers to the vertical movement of the external marker block mounted on the phantom surface, while tumor motion refers to the programmed internal motion of the embedded target in the superior-inferior (S–I), anterior-posterior (A–P), and lateral-medial (L–M) directions. Predictive 50 ms and 100 ms denote fixed prediction offsets applied to the surrogate motion to compensate for system latency.*

| **Type** | **Movement direction** | **Amplitude** (peak-2-peak) | **Cycle length** |
| --- | --- | --- | --- |
| Cos^6^ regular default | Surrogate Superior-inferior anterior-posterior lateral | 15 mm 15 mm 2 mm 2 mm | 5 s (12 bpm) |
| Cos^6^ regular fast | Surrogate Superior-inferior anterior-posterior lateral | 15 mm 15 mm 2 mm 2 mm | 3 s (20 bpm) |
| Cos^6^ regular slow | Surrogate Superior-inferior anterior-posterior lateral | 15 mm 15 mm 2 mm 2 mm | 10 s (6 bpm) |
| Cos^6^ regular small | Surrogate Superior-inferior anterior-posterior lateral | 5 mm 5 mm 1 mm 1 mm | 5 s (12 bpm) |
| Cos^6^ regular large | Surrogate Superior-inferior anterior-posterior lateral | 25 mm 25 mm 5 mm 5 mm | 5 s (12 bpm) |
| Cos^6^ regular predictive 50 ms | Surrogate Superior-inferior anterior-posterior lateral | 15 mm 15 mm 2 mm 2 mm | 5 s (12 bpm) |
| Cos^6^ regular predictive 100 ms | Surrogate Superior-inferior anterior-posterior lateral | 15 mm 15 mm 2 mm 2 mm | 5 s (12 bpm) |
| Cos^6^ regular predictive fast 50 ms | Surrogate Superior-inferior anterior-posterior lateral | 15 mm 15 mm 2 mm 2 mm | 3 s (20 bpm) |
| Cos^6^ regular predictive fast 100 ms | Surrogate Superior-inferior anterior-posterior lateral | 15 mm 15 mm 2 mm 2 mm | 3 s (20 bpm) |
| Cos^6^ irregular | Surrogate Superior-inferior anterior-posterior lateral | irregular | irregular |
| Patient  regular | Surrogate Superior-inferior anterior-posterior lateral | regular | regular |
| Patient irregular | Surrogate Superior-inferior anterior-posterior lateral | irregular | irregular |
